# 3D Virtual Modeling Realizations of Building Construction Scenes via Deep Learning Technique

**DOI:** 10.1155/2022/6286420

**Published:** 2022-03-31

**Authors:** Weihong Li

**Affiliations:** Xi'an University of Finance and Economics, Xi'an, Shanxi 710100, China

## Abstract

The architectural drawings of traditional building constructions generally require some design knowledge of the architectural plan to be understood. With the continuous development of the construction industry, the use of three-dimensional (3D) virtual models of buildings is quickly increased. Using three-dimensional models can give people a more convenient and intuitive understanding of the model of the building, and it is necessary for the painter to manually draw the 3D model. By analyzing the common design rules of architectural drawing, this project designed and realized a building three-dimensional reconstruction system that can automatically generate a stereogram (3 ds format) from a building plan (dxf format). The system extracts the building information in the dxf plan and generates a three-dimensional model (3 ds format) after identification and analysis. Three-dimensional reconstruction of architectural drawings is an important application of computer graphics in the field of architecture. The technology is based on computer vision and pattern recognition, supported by artificial intelligence, three-dimensional reconstruction, and other aspects of computer technology and engineering domain knowledge. It specializes in processing architectural engineering drawings with rich semantic information and various description forms to automatically carry out architectural drawing layouts. The high-level information with domain meanings such as the geometry and semantics/functions of graphics of the buildings can be analyzed for forming a complete and independent research system. As a new field of computer technology, the three-dimensional reconstruction drawings are appropriate for demonstrating the characteristics of architectural constructions.

## 1. Introduction

As the complexity of construction project management continues to increase, more and more automated and intelligent construction schedule methods have attracted attention from the traditional management field. However, the existing mainstream methods are subject to high cost and complex use restrictions and are difficult to apply to the complex construction schedule management scene. By comparing the characteristics of various 3D reconstruction technologies, a building construction schedule collaborative management automation system (DLR-P) based on deep learning 3D reconstruction technology was built. The system uses a high-speed camera to collect real-time image information of the construction site to complete the reconstruction from two-dimensional information to three-dimensional information and is combined with BIM dynamic model technology to achieve automatic control of the construction progress. Taking the construction site of a project in Banan District, Chongqing City as an example, the empirical study of the system was carried out, and various data during the operation of the system were verified and analyzed. The results show that the average 3D reconstruction time of the DLR-P system is 61*s*, which satisfies the basic schedule management requirements, can realize the automated management of the construction schedule, and effectively improves the efficiency of the construction schedule management. Compared with the existing management methods, it shows greater advantages in terms of operating costs and ease of use [1]. Construction schedule management of construction projects runs through the entire life cycle of construction [2].

**Figure 1:**
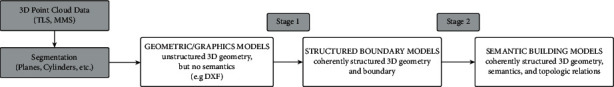
The deep learning framework with reconstruction technology.

Degree management efficiency is low, and it has caused cost overruns and legal disputes in many construction projects due to delays in construction schedules [3]. In order to achieve real-time, convenient, and economical construction schedule automation management, based on the existing intelligent construction field construction schedule automation management framework, a construction schedule collaborative management system framework based on deep learning 3D reconstruction technology is proposed, as shown in Figure 1, and describes the construction of the core part of the system's 3D reconstruction deep learning model and the system operation process [4, 5]. Regarding the topic of automated management of building construction schedules, scholars have conducted a lot of research in combination with various technologies. However, the existing research is difficult to apply to the complex practical requirements of building construction management [6]. The goal of the 3D reconstruction system of the building construction scene is to complete the whole parameter and whole process control of building construction, which integrates the basic functions. An important branch of artificial intelligence, known as deep learning, is so useful in smart building construction. Therefore, deep learning technology has extraordinary practical significance for the combination of artificial intelligence and the construction industry [7–9].

In recent years, the research and application of 3D reconstruction technology have been rapidly developed, but they still face many problems. To this end, this article will focus on the main progress and some representative research results of vision-based 3D reconstruction technology in recent years Introduce, provide reference for scientific research personnel, and on this basis, through comparison and analysis, explore the difficulties and hotspots in the research of 3D reconstruction technology, as well as possible development trends [10]. On the whole, the 3D reconstruction technology mainly uses the visual sensor to obtain the real information of the outside world and then obtains the 3D information of the object through the information processing technology or the projection model. In other words, the 3D reconstruction is a way of using 2D projection to restore the 3D information computer technology [11]. With the continuous advancement of science and technology, many research directions have emerged in 3D reconstruction technology, among which construction site construction is one of the main research directions of 3D reconstruction technology [12]. Some countries in Europe and the United States have already carried out research on 3D laser scanning technology. Long ago, Stanford University has carried out research on large-scale ground-mounted laser scanning systems and obtained more accurate experimental results [13]. Architectural technology refers to the methods and means to support the construction of construction projects, and the contextualization of architectural structures is the spatial intention of buildings. Among them, the architectural technology can convey the architectural intention. Besides, the actual construction effect of the building can be matched with the preconceived scene. In summary, the 3D modeling technology has been widely used in the field of architecture. Using 3D modeling technology to make architectural models, there is a realistic effect, in place. In addition, you can view the different effects of various parts of the building and the surrounding environment from a full range of angles. The innovation of this study lies in the adoption of 3 ds Max software as a development tool to complete the three-dimensional modeling and production of buildings, the production of topographic maps, the optimization of various scene models, and the final model output. structure. Therefore, the combination of the two can make the results of construction implementation have corresponding landing and controllability [14].

## 2. The Important Role of Digitalization in 3D Reconstruction of Buildings

At present, there are many research studies on building 3D data acquisition and building 3D reconstruction at home and abroad, but there are many differences in their methods. According to the source of the original data, they are mainly divided into the following three categories: based on satellite remote sensing images, laser point cloud, and tilt-based photography [15]. Three-dimensional building reconstruction techniques based on oblique photography are mostly used in nonprofessional fields. The viewfinder materials for oblique photography mainly include ground cameras and unmanned aerial photography. If the area is low and the density of the building is low, the ground camera is often used; if the building is taller and the building is dense, an unmanned camera can be used to shoot from the air. Use camera materials to take multiangle photographs of buildings and record the corresponding angles of the photographs. According to the relevant image processing and data processing algorithms, the three-dimensional architectural outline data of the building surface is calculated. In order to quickly obtain an accurate three-dimensional model of the construction site, it is necessary to obtain a three-dimensional model with high accuracy, high real-time performance, and strong anti-interference. The high precision of the 3D model is mainly evaluated from two directions: high-quality plane model and high-precision camera positioning. A high-quality plane model requires a three-dimensional model of the site which has rich texture information and is noise-free. This model should simulate the current three-dimensional scene with a continuous surface rather than a discrete point cloud and is less disturbed by external noise [16]. In addition, the most basic matching algorithm in the reconstruction algorithm is the nearest point iterative algorithm, but in practical applications, this algorithm is susceptible to interference from external factors. In addition, there are matching algorithms based on feature point extraction, such as SIFT algorithm, SURF algorithm, and ORB algorithm, but there is often error accumulation in the matching process. In addition to interframe matching based on the acquired data, there are also the realization of 3D reconstruction using global pose optimization, closed-loop detection, and beam adjustment, through image or key point-based relocation algorithms, which can reduce the matching error in the reconstruction process and external noise interference, thereby improving the accuracy of 3D model reconstruction [17].

The architectural space scene expression is the image display of the architectural space vision. Architectural construction is the practical process of implementing and constructing the scene. That is, the architectural space scene first uses the three-dimensional technology to present the scene-oriented planning and design and then use this planning and design to guide the actual construction, and the construction of the project brings the actual construction results closer to the expected effect in the design. Therefore, from architectural scene design to building construction is a connected process and an organic whole [18]. Architectural structure sceneization and building construction technology are complementary conceptual systems. The purpose of building construction technology application is to realize building structure sceneization. For example, building construction technology uses building materials to realize the relationship between architectural form design and architecture or landscape. It conveys the space effect and display effect that the building wants to express. At the same time, the sceneization of architectural structure can guide the construction of buildings through specific objects such as space display and landscape design. For example, the sceneization of existing architectural structures can inspire and even guide the construction of new architectural spaces [19]. In architectural design, the scene-based design of the building site is the embodiment of the construction results, and it is inseparable from the support of building construction technology. The different ways and themes of architectural scene expression will guide the reference of different construction techniques. For example, some scene space is relatively open, and it needs to be realized by combining corresponding construction technology and building materials in construction. For example, the content of the scene expression is comprehensive, and the building construction technology needs to be used to integrate the architectural space and the external environment of the building as a whole to form a unified scene space [20]. Deep learning is to simulate the visual mechanism of the human brain by combining low-level features to form more abstract high-level features or attribute categories to achieve complex function approximation and distributed representation of input data. The existing deep learning models mainly include convolutional neural networks, restricted Boltzmann machines, and deep belief networks. Deep learning is widely used in image recognition and behavior recognition, especially in the field of computer vision, which can realize the automatic recognition and classification of image scenes and the extraction of action gestures. Applying deep learning theories and methods can realize scene processing from the perspective of artificial intelligence, thereby effectively improving processing efficiency, which is explained as Figure 1 shows.

People generally abstract the specific spatial relationship of a set of specific objects as a scene. The constituent factors of the scene can be summarized in three aspects: (1) nonfixed characteristic factors refer to the actors in the scene with different characteristic attributes, showing different behavioral characteristics; (2) fixed characteristic factors refer to factors that are basically fixed or change little, such as floor construction characteristic environment; (3) semi-fixed characteristics refer to even in the same space. Different locations can produce and guide the occurrence of different behaviors, thereby forming different scenarios, such as pipelines and scaffolding [21]. Analyzed from the perspective of computer vision, the image semantics of construction scenes can be divided into three levels: low-level features, middle-level semantics, and high-level vision based on different levels of understanding. (1) The underlying features are low-level visual information, which can be directly obtained from the image, which is the most direct and objective description of the visual features of the image. (2) The middle-level semantics is represented by a visual packet model or semantic topics. The middle-level semantics is derived from the low-level visual features to express the feature information that is based on the content. (3) High-level vision refers to the semantic information obtained by people's high-level abstract cognition of images, which often contains higher-level and more abstract semantics than lower levels. There is a “semantic gap” between the low-level features and the high-level vision that people understand; that is, when judging image similarity, it is based on understanding the semantics of the description object, not just based on features such as the texture, color, and shape of the bottom layer.

## 3. Construction Site Management System Based on 3D Reconstruction Technology

### 3.1. System Framework

**Figure 2:**
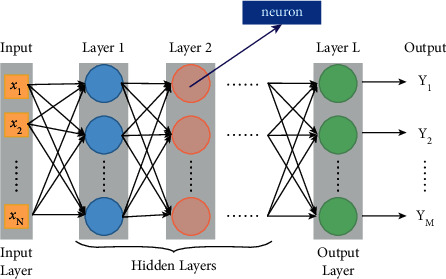
The logic diagram of the deep learning algorithm.

The framework of the construction progress collaborative management system based on deep learning 3D reconstruction technology consists of the following 4 parts to manage the closed-loop [22], as Figure 2 illustrated.

1. Construction site information: the construction site information is used as the frame. The basic data of the framework provides a management data basis for closed-loop management. The system uses high-speed camera sensors to collect 3D information on the construction site progress and then uses the 3D reconstruction deep learning model to realize the construction of a digital model of the construction site progress.
2. Three-dimensional reconstruction model of construction progress: based on real-time acquisition of multiview pictures, camera internal parameters, camera external parameters, and data matching information from the construction site, through features extraction, construction cost matching, depth estimation optimization, and point cloud model fusion technologies, the process obtains the actual 3D point cloud model of the construction site.
3. Ideal BIM construction schedule model: the model is the expected BIM construction schedule model (a 4D BIM model) jointly formulated by the design unit, the construction unit, and the owner before the construction of the building project, which includes not only the three-dimensional information during the construction process but also the progress information of the construction progress along with the time progress during the construction process. This part of the information has been formulated before the start of construction. During the construction process, this model is cross-compared with the point cloud model obtained in (2) to obtain construction progress difference information and then generate corresponding construction site resource adjustment opinions.
4. Construction site resource information: during the construction process, on-site management personnel, based on the construction site resource adjustment opinions generated in the process of (3), organize on-site labor, materials, machinery, and other resources to respond to achieve the purpose of on-site schedule adjustment, and after adjustment, the ideal BIM construction schedule model is dynamically adjusted to meet the overall schedule requirements.

### 3.2. 3D Reconstruction Deep Learning Model

The 3D reconstruction deep learning model is the core part of the system. The deep learning model used by the DLR-P system is MVSNet proposed by the Yaoyao team in 2018 [23]. This method is a classic three-dimensional reconstruction method proposed in recent years. While achieving good reconstruction results, it has also been used as an extension of the basic model to develop a series of deep learning models. Model principle: the depth information of different spatial positions is merged to construct the surface three-dimensional model information of the object. Model structure: the MVSNet model structure according to its functions mainly includes three parts: feature extraction, construction matching cost, depth estimation, and optimization.

**Figure 3:**
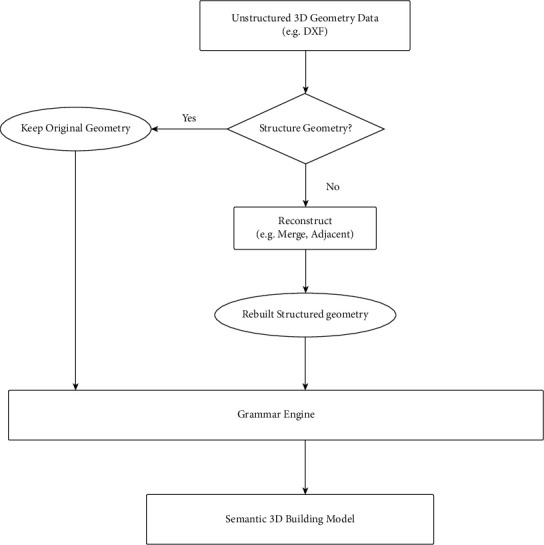
The algorithm hierarchy diagram.

(1) Feature extraction refers to image features extracted by a neural network. After the viewing angle is selected, the paired images, namely, the reference image and the candidate set, are input to the network model, an 8-layer two-dimensional convolutional neural network is used to extract the depth features of the stereopair, and the 32-channel feature map is output. In order to prevent the loss of semantic information after the input image is downsampled, the semantic information between the neighboring pixels of the pixel has been encoded into the 32-channel feature, and the network of each image extraction process is weight-sharing.
(2) Constructing the matching cost: the model uses the plane scanning algorithm to construct the matching cost of the reference image. After the feature extraction process, each image can obtain a corresponding feature map. According to the prior experience depth range, the reference image is scanned in the direction of its main optical axis, and the reference image is scanned from the minimum depth to the maximum according to a certain depth interval. Depth mapping: you can get a camera cone at different depth intervals, as shown in Figure 2. The feature maps in the candidate set are mapped to the camera cone. Through projection transformation, several images can form a corresponding number of feature bodies. This feature body is a representation of the matching cost. Finally, the cost accumulation of MVSNet is realized by constructing a three-dimensional structure composed of a cost map whose length and width are the same as the length and width of the reference image in the depth direction [24, 25].
As shown in the following formula:
  (1) $$\begin{array}{c} E\left(u,v\right)=\sum_{x,y}^{n}w\left(x,y\right){\left[I\left(x+u,y+v\right)-i\left(x,y\right)\right]}^{2}, \end{array}$$
where *u* and *v* are the offset
(3) Depth estimation and optimization: MVSNet's depth estimation is obtained by direct learning through deep neural networks. The network training method is to input the cost body and the corresponding true value of the depth map and use the SoftMax function to return the probability value of each pixel at the depth *θ* to complete the learning process from the cost to the depth value. The depth map and RGB image generated by the final model can be fused into a point cloud model. The algorithm hierarchy diagram is shown in Figure 3. When building the framework based on building information, this paper adopts the collaborative filtering algorithm to accelerate the data calculation speed, reduce the real-time response time, and enhance scalability. Thus, the accuracy, grammar, and real time of reconstruction levels are improved.

### 3.3. DLR-P System Operation Process

**Figure 4:**
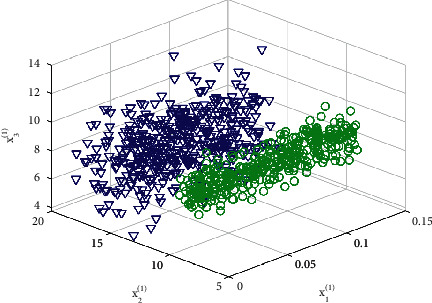
The data set with a noise feature of 3D information.

The main operating steps of the construction progress collaborative management system based on deep learning 3D reconstruction technology are as follows:

(1) Ideal BIM model construction: before running the DLR-P system, the construction should be constructed based on the project task objectives, engineering characteristics, and project environment. The collaborative design BIM model of the project greatly improves the efficiency of collaborative design of multiple specialties in a project at the same time. Besides, two-dimensional drawings are generated by direct projection and cutting of three-dimensional models, which greatly reduces the drawing workload of designers. The model should include the three-dimensional information of the project, expected progress information, expected cost information, labor demand information, material entry and exit information, and mechanical equipment demand information.
(2) Cooperative system construction: the system construction mainly includes two parts: an information collection module and a background processing module. The information collection module refers to the need to place the camera sensor in the required position of the construction site according to the requirements of different projects, and several sensors form an array to collect real-time appearance data of various construction site progress control targets. The background processing module includes a data processing part composed of a high-performance computer group and a progress management graphical part composed of a high-definition display. The communication between the above two modules is realized by a wireless local area network connection.
  (2) $$\begin{array}{c} \text{loss}=\frac{1}{2\text{m}}\sum {\left({\text{prediction}}^{\left(i\right)}-t^{\left(i\right)}\right)}^{2}. \end{array}$$
This formula represents the definition of the loss function.
(3) System operation: make sure that after each part of the system is built, link each part of the system in the same local area network environment, and set the image sensor capture angle and capture cycle. As the construction progress develops, ensure that the system runs in real time and that the sensor group can capture target images at different appropriate angles. The image data collected at the construction site is transferred to the background via the wireless network. Besides, the data set with a noise feature of 3D information is given in Figure 4. First, Colmap software is used to perform sparse reconstruction to calculate the camera pose matching information and other data, and then, the MVSNet 3D reconstruction deep learning model is used to generate the point cloud model of the corresponding scene. The system backstage imports the aforementioned point cloud model into the Revit software and the ideal BIM model for size comparison and compares and calculates the point cloud model and the ideal BIM model schedule according to the construction progress. Finally, the system outputs the current progress status and the corresponding construction site in a graphical display. Regulatory opinions can realize the fine management throughout the construction industry safety management [26–28].
  (3) $$\begin{array}{c} \text{loss}=\frac{1}{2m}\sum {\left({\text{prediction}}^{\left(i\right)}-t^{\left(i\right)}\right)}^{2},\\ L\left(p,u,t_{u},v\right)=L_{cls}\left(p,u\right)+\lambda \left[u\geq 1\right]L_{ioc}\left(t_{u},v\right), \end{array}$$
where N is the number of index categories, and *t* is the displacement in the logarithmic space.
According to the control opinions output by the DLR-P system, the personnel arrangement, material entry and exit, and the use of mechanical equipment for each relevant process on the construction site are uniformly deployed to optimize the construction schedule management.
(4) Model adjustment: input the active adjustment information such as personnel arrangement, material entry and exit, use of mechanical equipment on the construction site into the ideal BIM model, and adjust and optimize subsequent project construction work according to project requirements such as construction period and cost to form a system. The internal information of timely feedback is designed as a closed loop.

## 4. Image Semantic Extraction

**Figure 5:**
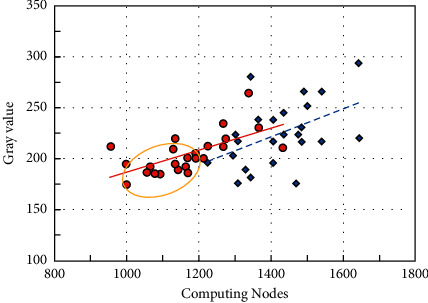
Comparison of experimental results of deep learning model.

Use CNN's deep learning structure to extract object semantics, including four processes of information input, preprocessing, feature extraction and selection, and classification decision learning. CNN can directly process two-dimensional images. Image feature extraction and dimensionality reduction are carried out step by step (Figure 5). The convolutional layer extracts image features through convolution kernels, the sampling layer reduces the dimensionality of image features, and the fully connected layer and classification layer are used for classification.

**Figure 6:**
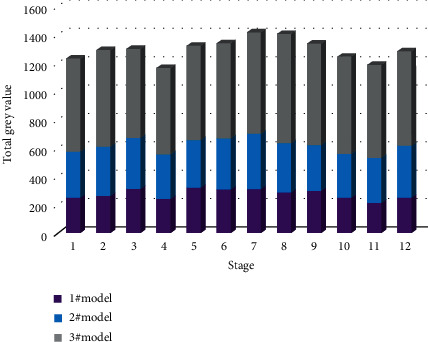
Histogram of gray values at different stages.

The histogram of gray values at different stages is given in Figure 6. In Figure 6, the target gray value on the y-coordinate represents the gray value of the reconstruction of the 3D building model expected by the model. Furthermore, the abscissa represents the different stages of the model refactoring process. The main implementation process of object semantic extraction is as follows:

(1) A 9 × 9 convolution kernel is used to convolve the input image, and the activation value of each neuron is mapped between −1 and 1 through the activation function tan*h*, and a total of 32 feature maps are output. Each output map may be the value of multiple input maps combined and convolved, specifically expressed as [29]
  (4) $$\begin{array}{c} X_{j}^{l}=f\left(\sum_{n\in M_{j}}X_{j}^{l-1}\times k_{nj}^{l}+b_{j}^{l}\right), \end{array}$$
where *f* is the activation function tan*h*; *X* is the output of the *j*-th image in the first layer; *M*_*j*_ is the set of input maps; *k* is one of the *j*-th images in the first layer and the *n*-th image in the 1-1 layer weight of the interval; *b* is the bias term of the *j*-th image in the first layer.
(2) Use the obtained 32 feature maps as the input of the second sampling layer, and perform dimensionality reduction processing. For this subsampling layer, there are 32 input maps and 32 output maps, and both dimensions are reduced to half of the original, which is specifically expressed as
  (5) $$\begin{array}{c} X_{j}^{l}=f\left(\text{down}\left(X_{J}^{l-1}\right)+b_{j}^{i}\right), \end{array}$$
where down represents the downsampling function.
(3) The output image of the second sampling layer is used as the input of the third convolution. Convolve the input image with a 9 × 9 convolution kernel, and use equation (1) to obtain 96 feature images. The coordinate relationship established by two related coordinate systems is as follows:
  (6) $$\begin{array}{c} u=\frac{x}{d_{x}}+u_{0},\\ v=\frac{y}{d_{y}}+v_{0}, \end{array}$$
where *u* and *vare* the axis corresponding to the two coordinate systems.
(4) The fourth subsampling layer again uses equation (2) to reduce the dimensions of the obtained convolutional features and obtain 96 10 × 10 feature maps, which are fully connected through the hidden layer and mapped to −1∼1 through the tank function between them. The logistic classifier is used for identification and classification, and the results are stored in the form of a variable to provide information support for the subsequent identification of the positional relationship between objects [30].
  (7) $$\begin{array}{c} x=\frac{fX_{c}}{Z},\\ y=\frac{fY_{c}}{Z}, \end{array}$$
where *X Y Z* is the coordinate of point *P*.

**Figure 7:**
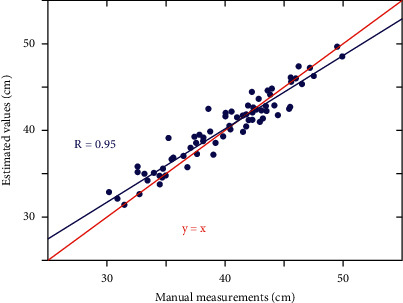
Error between calculation and actual measurement.

For the semantics of spatial relations, on the basis of object recognition, in order to locate each object area, the minimum edge rectangle method is used to approximate the area. The processed result is a series of rectangles, and each rectangle corresponds to a closed curve of the object contour. The error between calculation and the actual measurement is illustrated in Figure 7. The azimuth relationship between objects can be determined by establishing a directional relationship matrix.

## 5. Scene Data Processing

**Figure 8:**
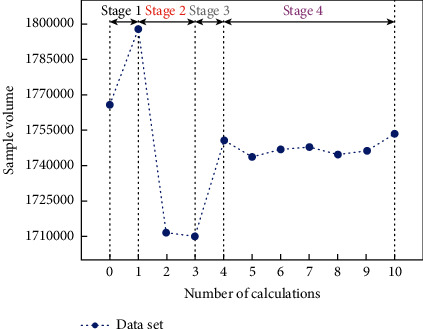
The curve of calculation times and sample error.

Scenario data can be expressed as the internal information of data mining from multiple scenarios and multiple dimensions, and the curve of calculation times and sample error is shown in Figure 8. Combined with existing research results, seven dimensions of unsafe behavior pan-scene data are determined from the perspective of manual collection, namely, time, location area, individual behavior, unsafe actions, nature of the behavior, traces of behavior, and level of risk. Unsafe state scenes can be described in two dimensions: scene objects and unsafe states. From the perspective of automated data collection, image semantic information is processed by multidimensional coding and relying on industry databases to form multidimensional pan-scene data. Obtained directly from digital devices, behavior traces and risk levels can be obtained from industry databases based on behavior semantics.

Formula (8) is used to quantify the change of the grayscale of the physical quantity of the local image.

(8) $$\begin{array}{c} E\left(x,y\right)=\sum w_{uv}{\left(I_{x+u,y+v}-I_{u,v}\right)}^{2}=\sum w_{uv}{\left[x\frac{\partial I}{\partial x}+\frac{\partial I}{\partial y}+o\left(x^{2}+y^{2}\right)\right]}^{2}. \end{array}$$

**Figure 9:**
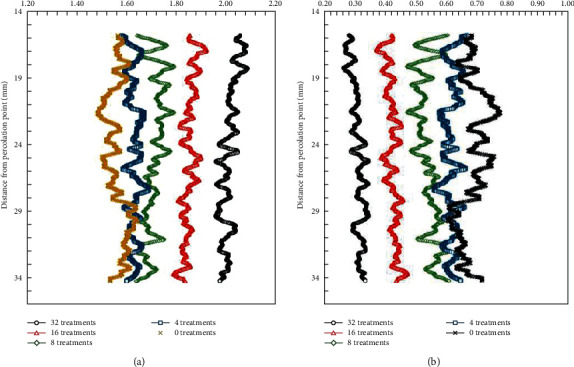
The average number of iterations and the median number of iterations. (a) The average number of iterations. (b) The median number of iterations.

Take the scenario of unsafe behavior-workers sleeping on a construction platform as an example, and specify the corresponding relationship: based on deep learning, the object semantics is that the worker wears a yellow helmet, and the corresponding behavior individual is the grassroots worker; spatial relationship semantics, and scene. The semantic common description is that the worker is on the construction bench, and the corresponding location area is the construction bench; the behavior semantics is that the employee sleeps on the construction bench, and the corresponding unsafe action is sleeping on the construction bench; based on the industry database, it is unsafe. The nature of the action behavior is an illegal action, the behavior trace is no trace, and the risk level is average. The average number of iterations and the median number of iterations are shown in Figure 9.

**Figure 10:**
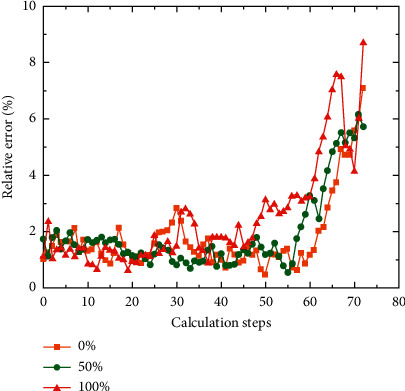
The value of mean square error predicted by the C-means clustering algorithm.

Applying deep learning methods to process construction site inspection pictures can identify unsafe human behaviors and unsafe conditions of objects. At the same time, it can process construction safety pan-scene data based on image semantics in terms of the application of construction safety pan-scenario data. As Figure 10 shows, the value of mean square error predicted by the C-means clustering algorithm of the physical quantity in local images fluctuates as the number of iterations.

(1) Statistical analysis of different dimensions can reflect the temporal and spatial distribution of unsafe behaviors, individual behavior classification, unsafe actions summary, and other internal knowledge, so as to provide enterprises with an accurate understanding of the characteristics of unsafe behaviors.
  (9) $$\begin{array}{c} F=U_{2}^{T}FU_{1}=U_{2}^{T}{\left[e\right]}_{v}U_{1}. \end{array}$$
Among them, the basic matrix *F* can be decomposed to obtain the transformation matrices *U*_1_ and *U*_2_,
(2) Explore the in-depth and multidimensional association rules of unsafe behaviors, and analyze when and what unsafe actions are prone to occur, where and what unsafe actions are prone to occur, and what kind of people are prone to what unsafe actions, so as to achieve personalized interventions on unsafe behaviors. Therefore, the 3D reconstructing technology can direct the process of architectural constructions in detail.

## 6. Advantages of Using 3D Reconstruction System in Building Construction Scenes

In the case study of the DLR-P system, the collaborative management method of construction progress based on deep learning 3D reconstruction technology showed the following three significant advantages:

(1) Automatic and efficient: compared with the traditional construction schedule management method that relies on manual patrol on the construction site, the proposed management method greatly improves the management efficiency and realizes the unmanned operation of the whole process, thereby reducing the labor intensity of the on-site management staff and achieving more objectiveness. The on-site progress monitoring refers to the management of the progress of each stage and the deadline of the final completion of the project in the process of project implementation. It is to draw up a reasonable and economical schedule within the specified time. The on-site progress monitoring eliminates various influencing factors in the manual management process.
  (10) $$\begin{array}{c} {\sum_{i=1}^{n}\left[\frac{u_{3}^{T}\left(p_{i}-p_{c}\right)}{u_{3}^{T}p_{c}}\right]}^{2}=\frac{u_{3}^{T}PPu_{3}}{u_{3}^{T}p_{c}p_{c}^{T}u_{3}}, \end{array}$$
where *λ* represents the scale factor, *w*_*i*_ is called the weight, snd *u*_3_ is the last line of *u*_*p.*_
(2) Cost economy: compared with the existing automatic management methods of “UAV + BIM” and “LiDAR + BIM,” the proposed management method greatly reduces the cost of system deployment and has higher promotion and practical value. In addition, while controlling the low cost of the system itself, this method can also improve the economic benefits of construction projects from the perspectives of saving labor costs, reducing rework waste caused by construction schedule factors, and optimizing the configuration of personnel, materials, and machinery.
  (11) $$\begin{array}{c} g\left(z\right)=\frac{t^{T}D^{-T}BD^{-1}t}{t^{T}t}. \end{array}$$
Among them, *A* is symmetric and positive definite, so *A* can be decomposed into *A* = *D*^*T*^*D*. Let *t* = *Dz*.
(3) Convenient application: the progress management of a construction project is a work that runs through the entire stage of the project construction. It has two important characteristics: long-term and dynamic. Therefore, the automated and intelligent way of realizing schedule management must be applicable to all kinds of changes in construction. Work in the scene: the automated schedule control system using high-speed cameras as the data collection method is more convenient to use than the method based on drones or LiDAR equipment in the process of project schedule management and control.

It is necessary to train drone operators and set up complex circulation routes in order to avoid collisions, and there is no need to consider the limitations of scanning instruments in special locations on the spot, which greatly liberates the information collection capabilities of the system and minimizes the schedule management work. The impact on various construction procedures is shown.

## 7. Conclusion

The 3D reconstruction technology based on deep learning shows good economic efficiency in the management of construction progress. Compared with the UAV method or lidar method in the current mainstream research, since only a high-speed camera is required as a sensor to collect data, it has significant advantages in terms of equipment cost, operating labor cost, and on-site coordination cost. The automation and intelligent management of building construction progress runs through the entire construction cycle, and the economic characteristics of this method can better meet the cost-sensitive characteristics of construction management units.

On the basis of extracting image object semantics, spatial relationship semantics, scene semantics, and behavior semantics, the corresponding relationship between semantic information and pan-scene data is constructed, and then through the industry unsafe behavior and unsafe physical state database, the automation of pan-scene data can be realized to deal with.

As the 3D reconstruction technology of building construction scenes is not very mature at present, many technical research studies are currently in the research stage. Due to various reasons, there has not been a widely used 3D reconstruction system of architectural drawings on the market so far. The 3D reconstruction system of architectural drawings realized by this system has some characteristics and some limitations. It can be further improved and perfected. It is hoped that a powerful, easy-to-operate, and widely used 3D reconstruction system of architectural drawings can be realized. Serving as a new technology, the three-dimensional drawings with applying reconstructing technology with the computer are appropriate for demonstrating the detailed geometric features of architectural constructions.

## Data Availability

The dataset can be accessed upon request.
